# Mechanisms in bradykinin stimulated arachidonate release and synthesis of prostaglandin and platelet activating factor

**DOI:** 10.1155/S096293519200022X

**Published:** 1992

**Authors:** D. Ricupero, L. Taylor, A. Tlucko, J. Navarro, P. Polgar

**Affiliations:** 1 Boston University School of Medicine Department of Biochemistry Boston MA 02118 USA; 2 Boston University School of Medicine Department of Physiology Boston MA 02118 USA

## Abstract

Regulatory mechanisms in bradykinin (BK) activated release of arachidonate (ARA) and synthesis of prostaglandin (PG) and platelet activating factor (PAF) were studied in bovine pulmonary artery endothelial cells (BPAEC). A role for GTP binding protein (G-protein) in the binding of BK to the cells was determined. Guanosine 5-O- (thiotriphosphate), (GTPτS), lowered the binding affinity for BK and increased the Kd for the binding from 0.45 to 1.99 nM. The Bmax remained unaltered at 2.25 × 10^-11^ mole. Exposure of the cells to aluminium fluoride also reduced the affinity for BK. Bradykinin-induced release of ARA proved pertussis toxin (PTX) sensitive, with a maximum sensitivity at 10 ug/ml PTX. GTPτS at 100 μM increased the release of arachidonate. The effect of GTPτS and BK was additive at suboptimal doses of BK up to 0.5 nM but never exceeded the levels of maximal BK stimulation at 50 nM. PTX also inhibited the release of ARA induced by the calcium ionophore, A23187. Phorbol 12-myristate 13-acetate or more commonly known as tetradecanoyl phorbol acetate (TPA) itself had little effect on release by the intact cells. However, at 100 nM it augmented the BK activated release. This was downregulated by overnight exposure to TPA and correlated with down-regulation of protein kinase C (PKC) activity. The down-regulation only affected the augmentation of ARA release by TPA but not the original BK activated release. TPA displayed a similar, but more potent amplification of PAF synthesis in response to both BK or the calcium ionophore A23187. These results taken together point to the participation of G-protein in the binding of BK to BPAEC and its activation of ARA release. Possibly two types of G-protein are involved, one associated with the receptor, the other activated by Ca^2+^ and perhaps associated with phospholipase A_2_ (PLA_2_). Our results further suggest that a separate route of activation, probably also PLA_2_ related, takes place through a PKC catalysed phosphorylation.


Mediators of Inflammation, 1, 133–140 (1992)

### Mechanisms in bradykinin stimulated arachidonate release and synthesis of prostaglandin and platelet activating factor

D. Ricupero,[1] L. Taylor,[1] A. Tlucko,[1] J. Navarro[2] and P. Polgar[1,CA]

Boston University School of Medicine, Departments of Biochemistry[1] and Physiology[2], Boston, MA 02118, USA

[CA] Corresponding Author

**REGULATORY mechanisms in bradykinin (BK) activated release of arachidonate (ARA) and synthesis of prostaglandin (PG) and platelet activating factor (PAF) were studied in bovine pulmonary artery endothelial cells (BPAEC). A role for GTP binding protein (G-protein) in the binding of BK to the cells was determined. Guanosine 5′-O-(thiotriphosphate), (GTPτS), lowered the binding affinity for BK and increased the Kd for the binding from 0.45 to 1.99 nM. The Bmax remained unaltered at $2.25 \times 10^{-11}$ mole. Exposure of the cells to aluminium fluoride also reduced the affinity for BK. Bradykinin-induced release of ARA proved pertussis toxin (PTX) sensitive, with a maximum sensitivity at 10 ug/ml PTX. GTPτS at 100 μM increased the release of arachidonate. The effect of GTPτS and BK was additive at suboptimal doses of BK up to 0.5 nM but never exceeded the levels of maximal BK stimulation at 50 nM. PTX also inhibited the release of ARA induced by the calcium ionophore, A23187. Phorbol 12-myristate 13-acetate or more commonly known as tetradecanoyl phorbol acetate (TPA) itself had little effect on release by the intact cells. However, at 100 nM it augmented the BK activated release. This was down-regulated by overnight exposure to TPA and correlated with down-regulation of protein kinase C (PKC) activity. The down-regulation only affected the augmentation of ARA release by TPA but not the original BK activated release. TPA displayed a similar, but more potent amplification of PAF synthesis in response to both BK or the calcium ionophore A23187. These results taken together point to the participation of G-protein in the binding of BK to BPAEC and its activation of ARA release. Possibly two types of G-protein are involved, one associated with the receptor, the other activated by $Ca^{2+}$ and perhaps associated with phospholipase $A_2$ ($PLA_2$). Our results further suggest that a separate route of activation, probably also $PLA_2$ related, takes place through a PKC catalysed phosphorylation.**

**Key words:** Arachidonate, Bradykinin, G-protein, Platelet activating factor, Prostaglandin synthesis

### Introduction

The exposure of endothelial cells to bradykinin (BK), a nonapeptide with inflammatory as well as vasoactive functions, results in the activation of a number of early metabolic events as determined in a number of cell types. These events include a biphasic increase in cytosolic $Ca^{2+}$,[1–4] a transient membrane hyperpolarization,[5] the activation of $K^+$ channels,[6] formation of diacylglycerol (DAG) and phosphatidic acid,[7] release of arachidonate (ARA) from phospholipid stores[8] and synthesis of prostaglandin (PG) and platelet activating factor (PAF).[9] Many of these events involve the activation of phospholipid related hydrolases including phospholipase $A_2$ ($PLA_2$) and phospholipase C (PLC).

Whereas the generation of inositol triphosphate ($IP_3$) and DAG are due to the activation of PLC, cumulative evidence suggests that the release of free arachidonic acid from phospholipid stores, at least in endothelial cells, involves $PLA_2$ activation by BK.[10] Although this release of fatty acid has been well described, the sequence of events which follows the binding of BK to the cell and results in the activation of $PLA_2$ remains to be fully elucidated. The resulting increase in cytosolic $Ca^{2+}$ is certainly a necessary step. However regulatory systems such as GTP-binding proteins (G-protein) and DAG activated protein kinase C (PKC) have

© 1992 Rapid Communications of Oxford Ltd.

been implicated in the process.[11,12] In this series of experiments we attempt to link some of these second messengers to elucidate the process of activation of $PLA_2$ by BK in the bovine pulmonary artery endothelial cell (BPAEC).

### Materials and Methods

*Cell culture:* Endothelial cells were isolated without the use of proteolytic enzymes. A freshly obtained calf pulmonary artery was cut open and lightly scraped with a scalpel. The resulting clumps of endothelial cells were placed into 35 mm dishes containing McCoy's 5A [Sigma, St Louis, MO] medium supplemented with 20% foetal bovine serum (FBS) [HyClone, UT].[13]

The homogeneity of endothelial cell cultures was determined morphologically and histochemically. At confluence the cells displayed a typical cobblestone appearance and stained positive for factor VIII antigen according to the method of Weinberg *et al.*[14] Endothelial cells were maintained in 25 $cm^2$ flasks containing 6 ml McCoy's 5A medium, 20% FBS, 50 $\mu$g/ml streptomycin and 50 units/ml penicillin. The culture medium was replaced every 3 days and the cells passaged biweekly. Cells to be used for experiments were passaged from 25 $cm^2$ flasks into 24-well plates, 20 $cm^2$ dishes or 60 $cm^2$ dishes at a density of 20 000 cells/$cm^2$ with trypsin (0.05%). The culture medium was replaced after 6 days. Two to 3 days after the last feeding, these cultures were used for the various experiments.

*Release of arachidonate:* Confluent cells in 24-well plates were prelabelled with $^3$H-arachidonate [79.9 Ci/mmol, New England Nuclear, MA] (0.2 $\mu$Ci per well) for 18 h. They were then washed once with McCoy's medium and preincubated for 2 h in McCoy's medium containing 1% FBS (0.5 ml per well). This process was found previously to increase the potential of the cell to synthesize prostaglandin.[13] In experiments with phorbol ester, 50 $\mu$l of a 10 × concentration of TPA was added to the wells during the last 10 min of the preincubation. Cells were then incubated in McCoy's medium containing 2 mg/ml bovine serum albumin (BSA) (Sigma, St Louis, MO, essentially fatty acid free from essentially globulin free) and the indicated additions. After 10 min the medium was removed and centrifuged (800 × *g*). Radioactivity was determined in a 200 $\mu$l aliquot of the supernatant.

*Radioimmunoassay (RIA) for prostaglandins:* Antibodies to 6-keto $PGF_{1\alpha}$ (6-K-$PGF_{1\alpha}$) were prepared in our laboratory. $PGI_2$ concentrations were determined as its stable degradation product 6-K-$PGF_{1\alpha}$. Cross-reactivity of the antisera against nontargeted PGs was less than 4%.[11] The radioimmunoassay was performed as described previously.[13]

*Platelet activating factor:* The synthesis of PAF was determined as described previously.[15] Generally the procedure of McIntyre *et al.* was followed.[9] Confluent cells (20 $cm^2$ Petri dishes) were incubated with $^3$H-acetate [3.6 Ci/mmol, New England Nuclear, Boston, MA] (50 $\mu$Ci/dish) in HEPES buffered Hank's balanced salt solution [Sigma, St Louis, MO], pH 7.4, with or without indicated additions. After 30 min at room temperature, the incubation was stopped by removing the medium and adding methanol/water (1:2) containing 50 mM acetic acid. The cells were scraped into this solution and transferred to screw-capped tubes containing chloroform. The plates were washed with methanol and the wash was added to the screw-capped tubes. After 2 h at room temperature, chloroform and water were added to split the monophase. The chloroform layer was dried under nitrogen and the lipids redissolved in small amounts of chloroform:methanol (2:1). The lipids were separated by thin layer chromatography (TLC) on alumina backed silica coated TLC plates in chloroform/methanol/acetic acid/water (25:14:4:2). Radioactivity was located by autoradiography and the radioactive spots were cut from the chromatogram and quantitated.

*Cell permeabilization:* In order that GTPτS [Boehringer Mannheim, IN] could penetrate the BPAEC, the permeability of the cells was increased by a 3 min exposure to saponin (0.02 mg/ml) in McCoy's medium at room temperature. The saponin [Sigma, St Louis, MO] was removed and GTPτS was added as indicated. This step was followed with vital stain.

*Binding of BK to cells:* The binding of BK to cells was carried out as described previously.[16] Binding was done in 24-well plates at 4°C for 2 h. The cultures were washed three times with 1 ml of phosphate-buffered saline (72 mM NaCl, 1.6 mM KCl, 5 mM $Na_2HPO_4$, 0.9 mM $KH_2PO_4$) at 4°C. This was followed by a 15 min equilibration with 0.5 ml of modified Hank's Balanced Salt Solution (HBSS), pH 7.3, containing 0.05% bovine serum albumin (BSA), 2 mM bacitracin, 10 mM HEPES, 120 mM *N*-methyl-D-glucamine (replacing NaCl), 0.65 mM $CaCl_2$, 0.25 mM $MgCl_2$ and 0.25 mM $MgSO_4$. This binding medium was removed and replaced with fresh, chilled binding medium containing the $^3$H-bradykinin [90 Ci/mmol, Amersham, IL]. Non-specific binding was determined in the presence of 3 $\mu$M unlabelled bradykinin. At the end of the incubation, the medium was aspirated and the cells were washed five times with 1 ml of the Modified Balanced Salt Solution with 0.2% BSA. This was followed by two washes with 1 ml of phosphate-buffered saline. Bound radioactivity was determined by solubilizing the cells with 0.5 ml of 0.2% sodium dodecyl sulphate. Radioactivity was quantitated in New England Nuclear [Boston, MA] 963 using an LKB Rackbeta Counter.

*Protein kinase C:* To determine PKC activity, cells were grown to confluence in 60 $cm^2$ dishes. They were treated with various effectors and then washed with cold PBS before harvesting by scraping into sample buffer (20 mM Tris buffer, pH 7.5 containing 0.33 M sucrose, 2 mM EDTA, 0.5 mM EGTA, and 100 $\mu$g/ml leupeptin). The scraped cells were centrifuged at 2 000 × ***g*** and the PKC was solubilized in 1% Triton × 100 in sample buffer for 30 min on ice. The insoluble material was removed by centrifugation and the supernatant containing the PKC was absorbed to DE52 resin. The resin was then washed with 10 ml sample buffer. PKC activity was eluted from the resin with sample buffer containing 100 mM NaCl. Activity was determined as described by Navarro *et al.*[17] Aliquots of the enzyme were mixed with 10 mM $MgCl_2$, 100 $\mu$M $\tau^{32}$P ATP (1 000 dpm/pmol), 50 $\mu$g of histone III-S with or without 1 mM $CaCl_2$, 5 $\mu$g of phosphatidylserine, and 20 ng of phorbol dibutyrate (PDBu) in a final volume of 50 $\mu$l. Samples were incubated for 10 min at 30°C. The reaction was stopped by spotting 25 $\mu$l of the reaction mixture onto Whatman 3MM paper and then washing the filter papers in 10% trichloroacetic acid containing 10 mM sodium pyrophosphate. Protein kinase C activity was determined by subtracting the amount of $^{32}$P incorporation into histone in the absence of added $Ca^{2+}$, phosphatidylserine, and PDBu.

*Assay for* sn-*1,2-diacylglycerol:* Diacylglycerol was determined according to the method of Preiss *et al.*[18] Cells were grown in 20 $cm^2$ plates and treated as indicated in the figure legends. Lipids were extracted and the dried lipids solubilized in 20 $\mu$l of an octyl-B-D-glycoside/cardiolipin solution (7.5% octyl-B-D-glycoside, 5 mM cardiolipin in 1 mM diethylenetriaminepentaacetic acid (DETAPAC) by sonication in a bath sonicator). Fifty microlitres reaction buffer (100 mM imidazole HCl, pH 6.6, 100 mM NaCl, 25 mM $MgCl_2$ and 2 mM EGTA), 2 $\mu$l 100 mM dithiothreitol, 10 $\mu$l diluted membranes containing DAG kinase (5 $\mu$g protein) and 8 $\mu$l water were then added. The reaction was started with the addition of 10 $\mu$l 10 mM ($\tau^{32}$P) ATP prepared in 100 mM imidazole, 1 mM DETAPAC, pH 6.6. After 30 min at 25°C the reaction was stopped with chloroform/methanol and the lipids extracted. The chloroform layer was washed twice with 2 ml 1% $HClO_4$. The volume of the chloroform layer was measured and an aliquot removed and dried under nitrogen for TLC. Silica Gel 60 thin layer chromatography plates were activated by running in acetone and air dried immediately before spotting samples. Plates were developed with chloroform:methanol:acetic acid (65:15:5), air dried and spots located by autoradiography. Radioactivity was quantitated by counting the scraped silica in a scintillation counter. The amount of *sn*-1,2-DAG present in the original sample was calculated from the sample volumes and the specific activity of the ATP.

### Results

*The effect of GTPτS on binding of BK to BPAEC:* To determine interaction between bradykinin, G-protein and phorbol 12-myristate 13-acetate (TPA) we first looked at the effect of GTPτS on the binding of BK to endothelial cells. This was done in intact cells which were permeabilized with saponin. A typical binding of BK to endothelial cells is illustrated as a binding curve (inset) and its Scatchard plot in Fig. 1. The permeabilization itself had no effect on the binding. The untreated cells bound with a Kd of 0.45 nM. Adding GTPτS (100 $\mu$M) to the incubation solution had a marked negative effect on the binding, increasing the Kd to 1.99 nM. Bmax stayed constant as $2.2 \times 10^{-11}$ mole. GDP$\beta$S, structurally similar to GTPτS but not an activator of G-protein, had no effect on binding (data not shown). In a separate experiment, aluminium fluoride, which dissociates the G-protein, reduced the binding of BK significantly as illustrated in Fig. 2. ATP (10 $\mu$M) had no effect.

*The effect of GTPτS on release of ARA:* As illustrated in Fig. 3, the release of arachidonate from endothelial cells preincubated with $^3$H-ARA is related to the GTPτS concentration up to approximately 100 $\mu$M. This release is time dependent as illustrated in Fig. 4. In this figure the release of arachidonate by GTPτS (100 $\mu$M) is compared to that by BK (50 nM). At 2 min after addition, BK more than doubles the release of label while GTPτS has little effect. Some effect by GTPτS is seen at 5 and 10 min, but this is small in comparison to BK. At 20 min after stimulation the effect by GTPτS is 70% that of BK.

The effect of pertussis toxin (PTX) was tested with regard to the release of arachidonate and PG synthesis. As illustrated in Fig. 5, PTX had a negligible inhibition of release by cells not treated with BK. The release caused by BK (50 nM) was approximately 12-fold the basal value. PTX reduced the BK activated increase of arachidonate release to approximately twice that of the basal value. Interestingly, PTX also blocked the release caused

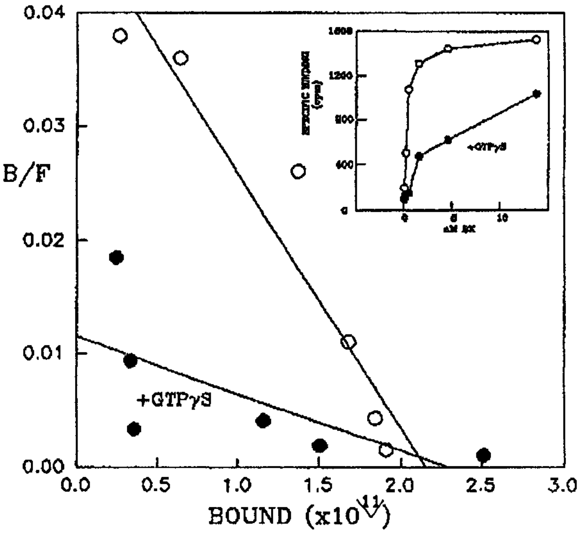

FIG. 1. The binding of BK to BPAEC: effect of GTPτS. Confluent cells were washed and incubated in medium containing 1% FBS for 2 h. They were permeabilized in McCoy's medium with saponin (0.02 mg/ml) for 3 min. The cells were incubated in fresh medium with or without GTPτS (100 μM) for 10 min. After this incubation, the cells were rapidly chilled by placing on ice and washing with ice cold phosphate buffered saline. Binding was performed as described in the methods section. Scatchard plot was generated using the Ligand program. ○ = specific binding (total binding—nonspecific); ● = specific binding in presence of GTPτS.

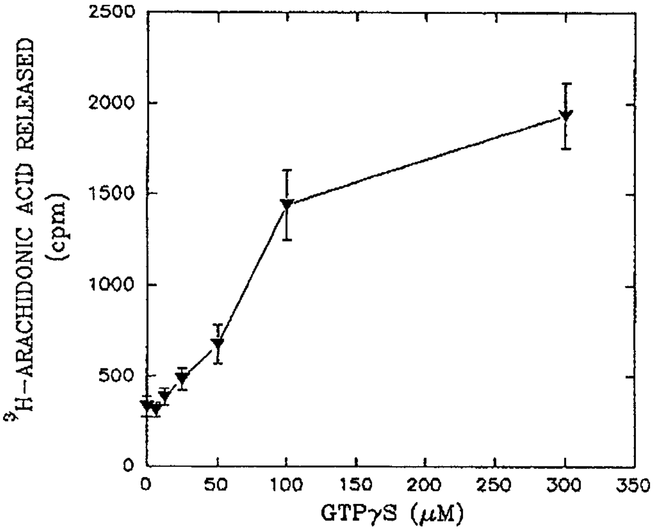

FIG. 3. Concentration related effect of GTPτS on the release of ARA. Confluent cells were labelled with $^{3}$H-arachidonate for 18 h. Cells were then washed and incubated in medium containing 1% FBS for 2 h. Cells were permeabilized by incubation with McCoy's medium plus saponin (0.02 mg/ml) for 3 min. The cells were washed and incubated in medium containing 2 mg/ml BSA and the indicated concentrations of GTPτS. After 15 min medium was removed and arachidonate release determined as described in the methods section. Results are the mean of quadruplicate cultures.

by A23187 to a similar degree. The effect was related to the PTX concentration as illustrated in Fig. 6. Some effect is seen at 1 ng/ml; by 10 ng/ml maximum effect is seen. No further inhibition is detected at 100 ng/ml.

The interaction between BK and GTPτS on the release of arachidonate is illustrated in Fig. 7. GTPτS alone at 100 μM increased release. BK increased release from 0.5 nM up to 5 nM. At suboptimal concentrations of BK, the addition of GTPτS increased release above that of BK up to 0.5 nM BK. Above this concentration of BK GTPτS had no additive effect.

*Phorbol 12-myristate 13-acetate:* Exposure of BPAEC to TPA (100 nM) for 10 min in absence of BK had no effect on the release of arachidonate (Fig. 8). However, as illustrated in the same figure, TPA increased BK stimulated release. When the cells were treated overnight with TPA (500 nM) to down-regulate PKC activity and then were pretreated with fresh TPA and exposed to BK the

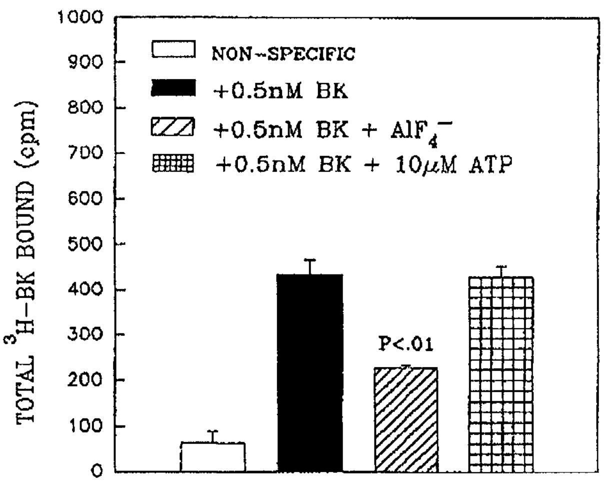

FIG. 2. Effect of aluminum fluoride on BK binding to BPAEC. Confluent cells were washed and incubated in medium containing 1% FBS for 2 h. They were then placed on ice and washed with ice-cold phosphate buffered saline. Sodium fluoride (25 mM) and aluminium chloride (10 μM) were added in cold binding buffer containing $^{3}$H-bradykinin (0.5 nM) plus or minus unlabelled bradykinin (3 μM). Binding was determined as described in the methods section.

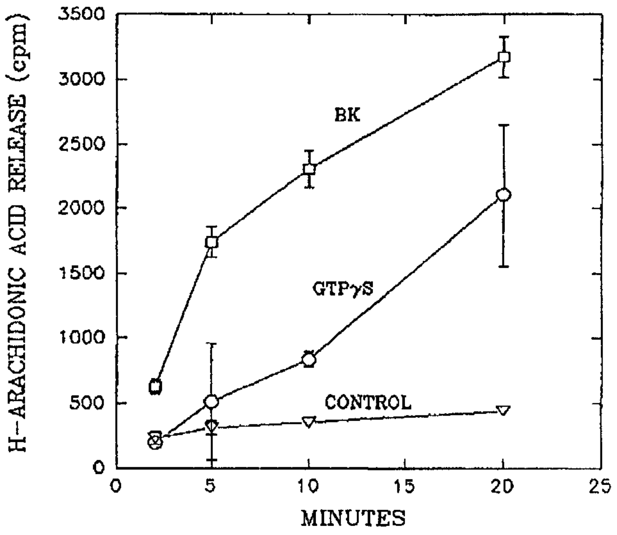

FIG. 4. Time curve of BK and GTPτS effected release of ARA. Cells were treated as described in Fig. 3 except that medium was harvested at the indicated times after bradykinin (50 nM) or GTPτS (100 μM) addition.
□ = BK treated
○ = GTPτS treated
△ = Non-stimulated release

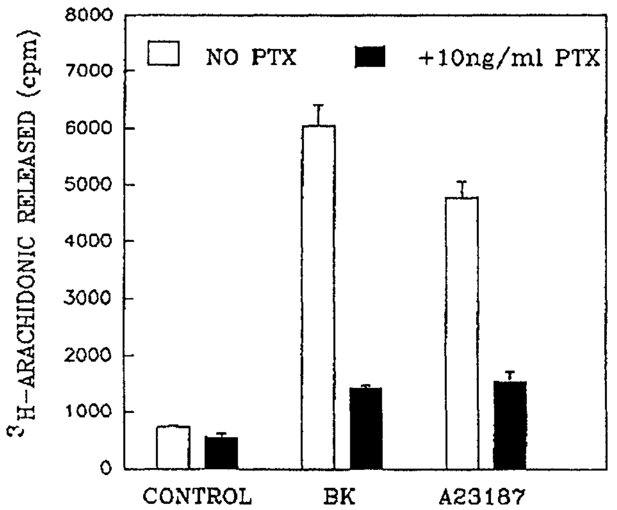

FIG. 5. The effect of PTX on BK and A23187 stimulated release of ARA. Confluent cells were labelled with $^3$H-arachidonate for 18 h. The cells were washed and incubated in medium containing 1% FBS with or without pertussis toxin (10 ng/ml). After 4 h the medium was removed and medium containing BSA (2 mg/ml) with or without BK (50 nM) or A23187 (10 $\mu$M) was added. After 15 min, medium was harvested from quadruplicate wells and radioactivity determined. Results are the means of quadruplicate cultures.
□ = Cells not treated with PTX
■ = Cells treated with PTX

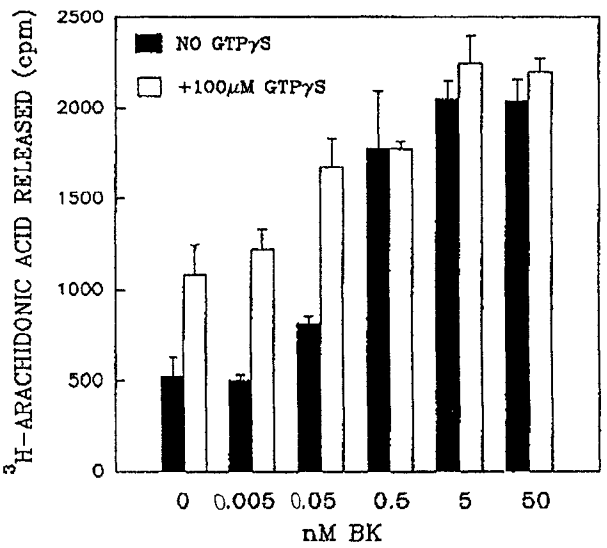

FIG. 7. Synergism between BK and GTPτS in the release of ARA. Cells were treated as described in Fig. 3 except that they were incubated with the indicated concentrations of BK plus or minus GTPτS (100 $\mu$M).
■ = Only BK at 0 to 50 nM
□ = BK at 0 to 50 nM + 100 $\mu$M GTPτS

response to BK remained unaltered, but no augmentation by TPA was observed (Fig. 8). Determination of PKC activity, as illustrated in Fig. 8 (insert) shows that PKC was indeed down-regulated by exposure of the cells to 500 nM TPA overnight. In Fig. 9 a similar effect by TPA is seen in BK activated PAF synthesis. In this case TPA at 16 nM increased BK-activated PAF production by approximately three-fold. Interestingly the response to A23187, a calcium ionophore, was also augmented by TPA.

As a further illustration of the participation of PKC in BK activated release, Fig. 10 shows that in response to BK, BPAEC produce DAG. DAG

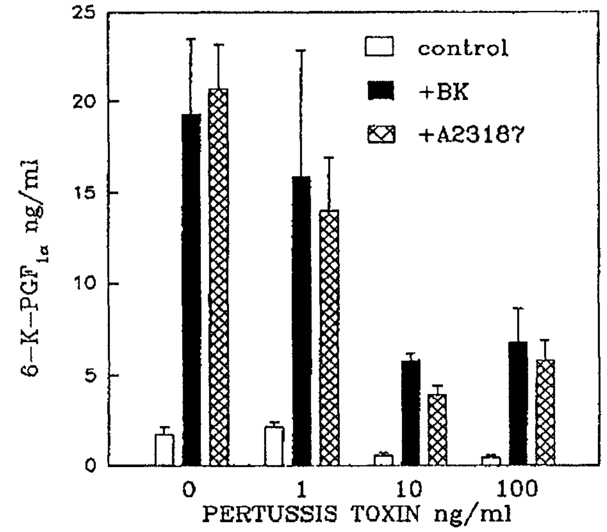

FIG. 6. Inhibitory effect of PTX at various concentrations. Confluence cells were washed and incubated in medium containing 1% FBS and the indicated concentration of pertussis toxin (PTX). After 4 h this medium was removed and the cells incubated in McCoy's medium with or without bradykinin (50 nM) or ionophore A23187 (10 $\mu$M). After 20 min medium was harvested from quadruplicate wells and assayed for 6-keto-PGF$_{1\alpha}$ content by RIA.
□ = Control, untreated cells
■ = BK treated cells
⊠ = Ionophore A23187 treated cells

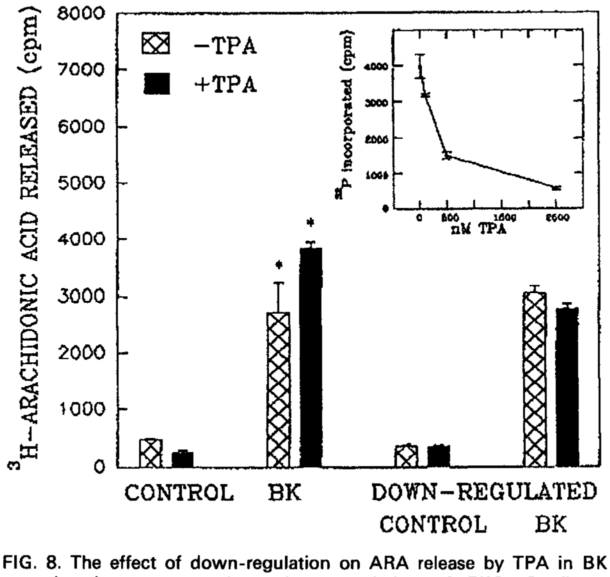

FIG. 8. The effect of down-regulation on ARA release by TPA in BK treated cultures, concomitant down-regulation of PKC. Confluent cultures were labelled with $^3$H-arachidonate for 18 h. For PKC down-regulation TPA (500 nm) was included during this 18 h incubation. The cells were washed and the medium changed to medium containing 1% FBS (0.5 ml/well). After 2 h 50 $\mu$l of a 1 $\mu$M solution of TPA was dropped into the appropriate wells. After 10 min the medium was removed and medium containing 2 mg/ml BSA with or without BK (50 nM) was added. After 10 min this medium was collected and arachidonate release determined as described in the methods section. Results are the means of quadruplicate cultures. Insert: Parallel cultures were set up in 60 cm$^2$ Petri dishes. They were treated with the indicated concentrations of TPA for 18 h. PKC activity was determined as described in the Methods section.
⊠ = Cultures not treated with TPA
■ = Cultures treated with 100 nM TPA

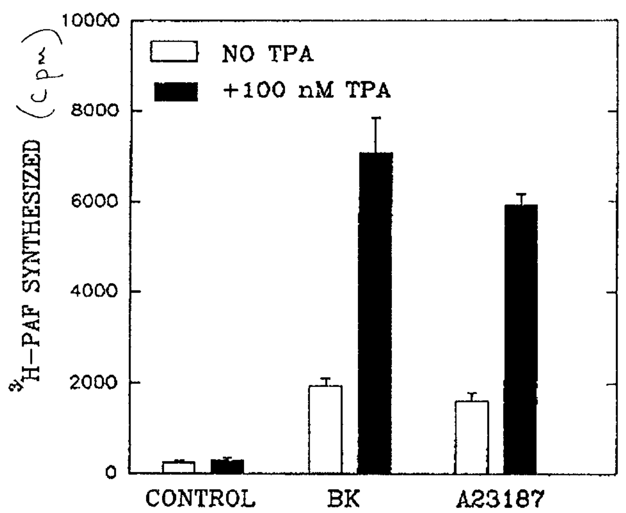

FIG. 9. Effect of TPA on BK stimulated PAF synthesis. Confluent cells in 20 cm² Petri dishes were treated with the indicated concentrations of TPA for 10 min. The cells were washed and assayed for PAF production in response to BK (50 nM) or calcium ionophore (10 μM) as described in the methods section.

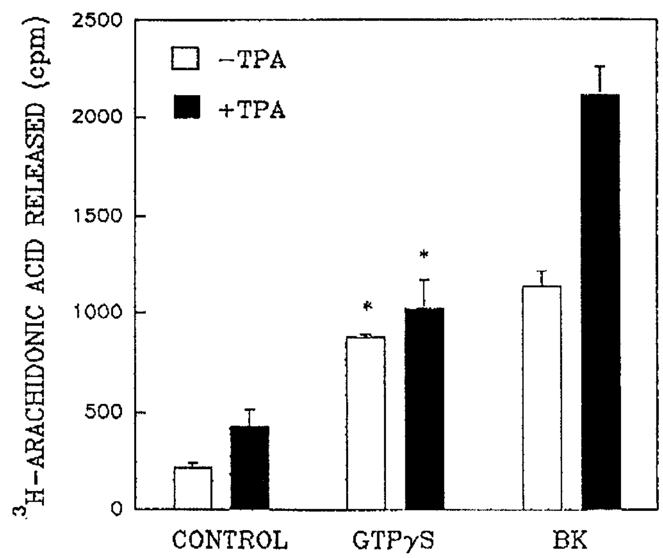

FIG. 11. The effect of TPA on GTPτS and BK stimulated release of ARA. Cells were treated as described in Fig. 9. After the 10 min PMA incubation, cells were permeabilized with saponin (0.02 mg/ml) for 3 min and then medium containing 2 mg/ml BSA with or without BK (50 nM) or GTPτS (100 μM) was added. After 15 min the medium was collected and ³H-arachidonate release determined as described in the methods section. Results are the means of quadruplicate cultures.
□ = Cultures treated with either (nothing, GTPτS or BK).
■ = Culture treated as above + 100 nM TPA.

production was determined at various concentrations of BK from 0.01 to 50 nM. In this case a maximum was reached between 0.5 and 2 nM BK.

The interaction of TPA with BK and GTPτS is illustrated in Fig. 11. The release of arachidonate was determined with either BK or GTPτS in the presence or absence of TPA. In this case TPA basically doubled the effect of BK. It proved to have no effect on GTPτS stimulated release.

### Discussion

In the experiments described we investigated the high affinity $B_2$ binding site of BK in intact, viable endothelial cells. We found evidence in these cells of only one high affinity $B_2$ site.[16] Results illustrated in Figs 1 and 2 show that this binding of BK involves G-protein. The addition of GTPτS, a non-hydrolysable analogue of GTP, to cells previously exposed to saponin increased the Kd of binding by approximately four-fold (0.45 nM to 1.99 nM) while the $B_{max}$ remained unchanged at $2.25 \times 10^{-11}$ mole. This suggests dissociation of G-protein leading to a lower affinity for BK. GTPτS is known to maintain the G-protein in a permanently dissociated state. Exposure of the cells to aluminium fluoride, which dissociates G-protein, also lowered the binding of BK to the BPAEC. These experiments, conducted at the cellular level with the receptor intact, are consistent with reports using plasma membranes isolated from fractionated myometrium[19,20] and the bovine aorta.[21] Our results are also consistent with the recent report of the cloning of the $B_2$ receptor for BK from rat uterus as a typical G-protein coupled receptor.[22]

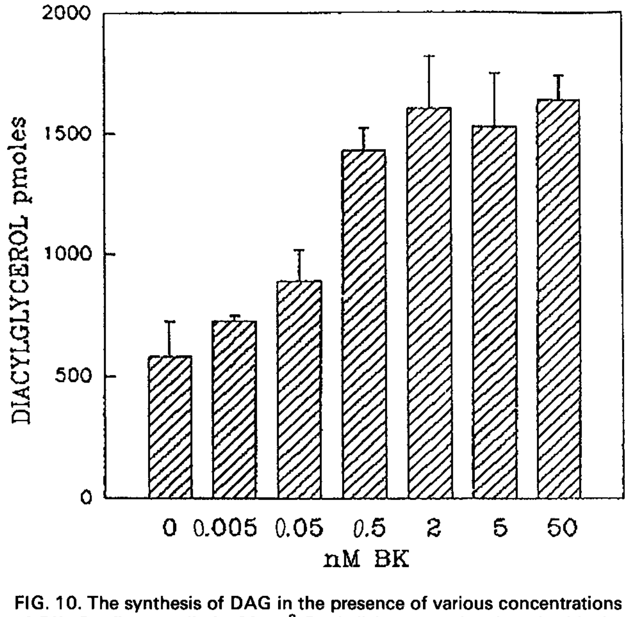

FIG. 10. The synthesis of DAG in the presence of various concentrations of BK. Confluent cells in 20 cm² Petri dishes were incubated with the indicated concentrations of BK for 10 min. DAG was determined as described in the methods section.

Separate G-proteins appear to function in the regulation of $PLA_2$ and PLC. A number of reports suggest that the generation of inositol triphosphate ($IP_3$) is not PTX sensitive. In contrast, the action of $PLA_2$ is PTX sensitive. For example, in 3T3 fibroblasts, thrombin and BK increase $Ca^{2+}$ influx, arachidonate release and $IP_3$ release. PTX was reported to inhibit arachidonate release but not $IP_3$ release.[23] In FRTL5 thyroid cells, adrenergic $a_1$ receptors are coupled to $PLA_2$ by a PTX sensitive G-protein, and to PLC by a PTX insensitive G-protein.[24]

The generation of $IP_3$ in BPAEC in response to BK has been shown to be G-protein linked but not sensitive to PTX.[25] Results here show a clear inhibition of ARA release by PTX in the same cells. Our observations further illustrate that PTX inhibits not only BK-activated release but also A21387-activated release. This suggests that the PTX sensitive G-protein may be functioning beyond the $Ca^{2+}$ mobilization step. Nakashima *et al.* reported previously that *N*-formyl-methionyl-leucyl-phenylalanine (fMLP) activated neutrophil release of arachidonate and PG synthesis.[26] They also reported that G-protein is involved in this interaction. They proposed that in the neutrophil G-protein lowers the requirement of $PLA_2$ for $Ca^{2+}$.

One explanation for these observations is that the PTX insensitive G-protein is associated directly with the BK receptor while the PTX sensitive G-protein is perhaps associated directly with $PLA_2$. The G-protein associated with the receptor may be involved in the BK regulation of cytosolic $Ca^{2+}$ concentrations. G-protein was recently shown to participate in the regulation of receptor operated $Ca^{2+}$ channels in platelets.[27] Also, a recent report illustrated that the complex $AlF_4^-$ activated calcium influx in endothelial cells.[7] The BK receptor may form a part of a $Ca^{2+}$ transporter.[28] The PTX sensitive G-protein in BPAEC may be one of the subtypes of $G_i$. Clark *et al.*[29] have identified a 41 kDa protein as ADP ribosylated by PTX, consistent with the molecular weight of $G_i$. Lee *et al.* have demonstrated that endothelial cells express mRNA for all three subtypes of $G_i$.[30]

Our results illustrate that TPA is also involved in the release of arachidonate in BPAEC. Generally phorbol ester has been shown to inhibit the action of PLC in the generation of $IP_3$.[31] This inhibition is related to PKC. With regard to the activation of $PLA_2$ by phorbol ester, the literature is more tenuous. For example, in the experiments using MDCK cells,[31] TPA itself stimulated the release of arachidonic acid which was inhibited by 1-(5-isoquinolinylsulphonyl)-2-methyl piperazine (H7), an inhibitor of PKC. In another report using MDCK cells, the BK response was only slightly sensitive to PKC inhibitors (sphingosine, H7, staurosporine).[32] The BK-stimulated release of arachidonic acid could not be enhanced by TPA in PKC down-regulated BPAEC. However, in these cells TPA itself stimulated the release of arachidonate which was attenuated (50%) by PKC inhibitors.[32] Phorbol ester was reported to augment BK-stimulated PG synthesis in other cell types such as Swiss 3T3.[33] Other effectors such as thrombin, which activates PG and PAF synthesis in human endothelial cells, are augmented by TPA.[34] In the described experiments, TPA alone did little in the absence of BK. However, in the presence of BK the release of arachidonate was synergistic. This synergism by phorbol was abolished when PKC was down-regulated by incubating the cells overnight with TPA. However, the BK-stimulated release was totally unaffected by the down-regulation of PKC. This action by TPA appears related to $PLA_2$. This is suggested in Fig. 9 by the TPA-augmented, BK-induced synthesis of PAF. In these experiments the synthesis of PAF was determined through the remodelling pathway which utilizes $PLA_2$ to remove fatty acid from the *sn*-2 position of 1-*O*-alkyl-2-acyl-*sn*-glyceryl-3-phosphocholine and incorporates acetate into the lyso-PAF via lyso-PAF acetyl-CoA acetyltransferase (LPAT) to form the 1-*O*-alkyl-2-acetyl-*sn*-glyceryl-3-phosphocholine (PAF). It is possible that TPA in conjunction with BK also activates LPAT. This is suggested by the considerably larger augmentation by TPA of BK-stimulated PAF synthesis than of ARA release. In fact Heller *et al.*[35] reported recently that thrombin-activated PAF synthesis in human umbilical vein endothelial cells involved the activation of LPAT. TPA alone did not activate LPAT. The effect of thrombin plus TPA on LPAT activity was not determined.

TPA also did not affect GTPτS activation of release (Fig. 11), suggesting that the two events represent separate paths to the activation of $PLA_2$. As we illustrated, BK does generate DAG (Fig. 10), and thus may, under certain conditions, additionally activate the release of arachidonate through the action of protein kinase C.

### References

1. Colden-Stanfield M, Schilling WP, Ritchie AK, Eskin SG, Navarro LT, Kunze DL. *Circ Res* 1987; **61:** 632–640.
2. Schilling WP, Ritchie AK, Navarro LT, Eskin SG. *Am J Physiol* 1988; **255:** H212–H227.
3. Johns A, Lategan TW, Lodge NJ, Ryan US, Van Breemen C, Adams DJ. *Tissue Cell* 1987; **19:** 733–745.
4. Buchan KW, Martin W. *Br J Pharmacol* 1991; **102:** 35–40.
5. Mehrke PU, Daut J. *J Physiol (Lond)* 1991; **439:** 277–299.
6. Thuringer D, Diarra A, Sauve R. *Am J Physiol* 1991, **261:** H656–H666.
7. Graier WF, Schmidt K, Kurovetz WR. *Cell Signal* 1990; **2:** 369–375.
8. Hong SL, Deykin D. *J Biol Chem* 1982; **257:** 7151–7154.
9. McIntyre TM, Zimmerman GA, Satoh K, Prescott SM. *J Clin Invest* 1987; **76:** 271–280.
10. Kaya H, Patton GM, Hong SL. *J Biol Chem* 1989; **264:** 4972–4977.
11. Burch RM. *FEBS Lett* 1988; **234:** 283–286.
12. Burch RM. *Mol Neurobiol* 1989; **3:** 155–171.
13. Menconi M, Hahn G, Polgar P. *J Cell Physiol* 1984; **120:** 163–168.
14. Weinberg KS, Douglas WHJ, McNamee DR, Lanzillo JJ, Fanburg BL. *In Vitro* 1985; **18:** 400–406.
15. Heinson C, Polgar P, Fishman J, Taylor L. *Arch Biochem Biophys* 1987; **257:** 251–258.
16. Cahill M, Fishman JB, Polgar P. *Agents and Actions* 1988; **24:** 224–229.
17. Navarro J. *J Biol Chem* 1987; **262:** 4649–4652.
18. Preiss J, Loomis CR, Bishop WR, Stein R, Niedel J, Bell RM. *J Biol Chem* 1987; **262:** 4649–4652.
19. Liebman C, Offermanns S, Hinsch KD, Schnittler M, Morgat JI, Reissmann S, Schultz G, Rosenth W. *Biochem Biophys Res Comm* 1990; **167:** 910–917.
20. Leeb-Lundberg LM, Mathis SA. *J Biol Chem* 1990; **265:** 9621–9627.
21. Keravis TM, Nehlig H, DeLacroix MF, Regoli D, Hiley CR, Stoclet JC. *Eur J Pharmacol* 1991; **207:** 149.
22. McEachern AE, Shelton ER, Bhakta S, Obernolte R, Bach C, Zuppan P, Fujisaki J, Aldrich RW, Jarnagin K. *Proc Natl Acad Sci USA* 1991; **88:** 7724–7728.
23. Murayama T, Ui M. *J Biol Chem* 1985; **260:** 7226–7233.

24. Burch RM, Luini A, Axelrod J. *Proc Natl Acad Sci USA* 1986; **83**: 7201–7206.
25. Voyno-Yasenetskaya TA, Tkachuk VA, Cheknyova EG, Panchenko MP, Grigorian GY, Vavrek RJ, Stewart JM, Ryan US. *Faseb J* 1989; **3**: 44.
26. Nakahima S, Nagata K, Ueeda K, Nozawa Y. *Arch Biochem Biophys* 1988; **261**: 375–383.
27. Bochkov VN, Feoktistov IA, Avdonin PV, Tkachuk VA. *Biokhimiia* 1989; **54**: 1533–1542.
28. Weiss C, Atlas D. *Brain Res* 1991; **543**: 102–110.
29. Clark MA, Conway TM, Bennet CF, Crook ST, Sudei JM. *Proc Natl Acad Sci USA* 1986; **83**: 7320–7324.
30. Lee RT, Brock TA, Tolman C, Bloch KD, Siedman JG, Neer E. *FEBS Lett* 1989; **249**: 139–142.
31. Portilla D, Mordhorst M, Bertrand W, Morrisson AR. *Biochem Biophys Res Comm* 1988; **153**: 454–459.
32. Weiss BA. *Am J Pharm and Exp Ther* 1989; **36**: 317–326.
33. Burch RM, Lin MA, Axelrod J. *J Biol Chem* 1988; **263**: 4764–4767.
34. Zavoico GB, Hrbolich JK, Gimbrone MA Jr., Schafer AI. *J Cell Physiol* 1990; **143**: 596–605.
35. Heller R, Bussolino F, Ghigo D, Garbarino G, Pescarmona G, Till U, Bosia A. *J Biol Chem* 1991; **266**: 21358–21361.

ACKNOWLEDGEMENTS. This work was supported by NIH Grants HL25776 and AG00115.

**Received 6 February 1992; accepted in revised form 25 February 1992**
